# Classical Concept of Semiconductor Heterojunctions in the Approach to Nanohybrid Catalysts

**DOI:** 10.1021/acsami.4c08595

**Published:** 2024-07-11

**Authors:** Jacek Tyczkowski, Hanna Kierzkowska-Pawlak

**Affiliations:** Department of Molecular Engineering, Faculty of Process and Environmental Engineering, Lodz University of Technology, Wolczanska 213, 93-005 Lodz, Poland

**Keywords:** nanocatalyst design, heterojunctions, nanohybrids, thin films, cold plasma deposition

## Abstract

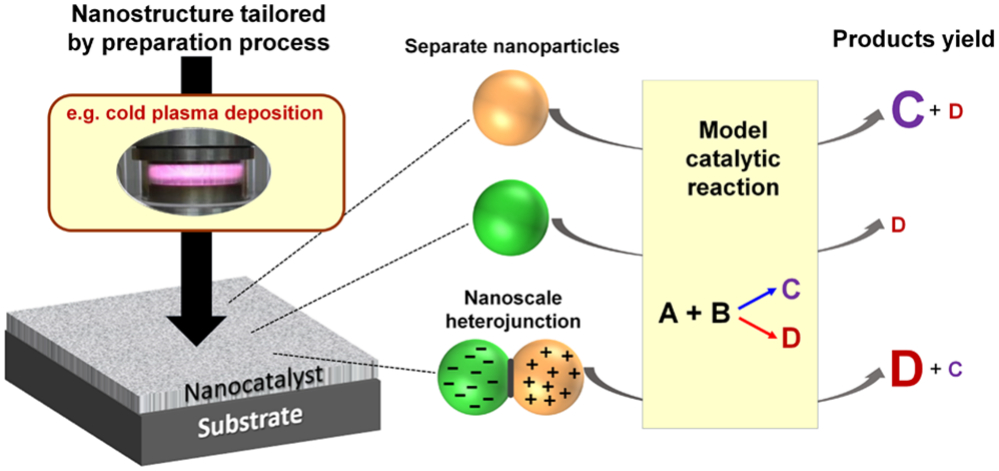

Recalling the well-established theory of heterojunction formation between two different semiconductors or a semiconductor and a metal can elucidate the remarkable catalytic properties of nanohybrid systems employed in thermal catalysis. Upon the creation of heterojunctions, involved nanoparticles or nanometer-sized thin films, as a result of their dimensions, may become entirely filled with space charges generated from the development of depletion or accumulation regions. This phenomenon dictates the nature of catalytic sites and consequently affects the catalytic activity of such nanohybrids. The following perspective presents this concept and examples of experimental results that substantiate its validity, along with an extremely effective tool, cold plasma deposition, for designing and realizing in a controlled manner the structure of nanohybrids with heterojunctions. This approach will undoubtedly broaden the view of the contemporary “alchemy” of nanocatalysts.

## Introduction

Despite the tremendous advances in catalyst research, including the use of *operando* techniques that examine catalysts while they work or computational chemistry methods now used to screen hypothetical catalysts across material space on an impressively large scale, the pursuit of new dedicated catalytic structures is still more of a “trial and error” approach than a fully rational design strategy. In the field of thermal heterogeneous catalysis, combinations of different active phases are frequently employed, typically in nanostructured form (sometimes referred to as nanohybrid structures), but these are created for a given catalytic process in a mysterious way, similar to alchemy.^1^ The aspiration of achieving catalysts through deliberate design has been a major goal of the catalysis community for years.^2^

The interaction of different materials in complex nanostructures is usually interpreted as a “synergistic effect”, which includes changes in active phase dispersion, surface properties, electronic structure, etc., thus everything that could favorably influence the observed catalytic properties.^3,4^ Unfortunately, a thorough understanding of the interaction of different materials in nanohybrid structures and the mechanisms of catalytic reactions involving such structures is still lacking.

Some light on the aforementioned problem could be shed by the concept of generating space charges as a result of the formation of heterojunctions between nano-objects, of which the sizes are comparable to the sizes of the emerging space charge regions. The presence of such regions may explain many hitherto incomprehensible effects on the surfaces of nanocatalysts.^5^

**Figure 1:**
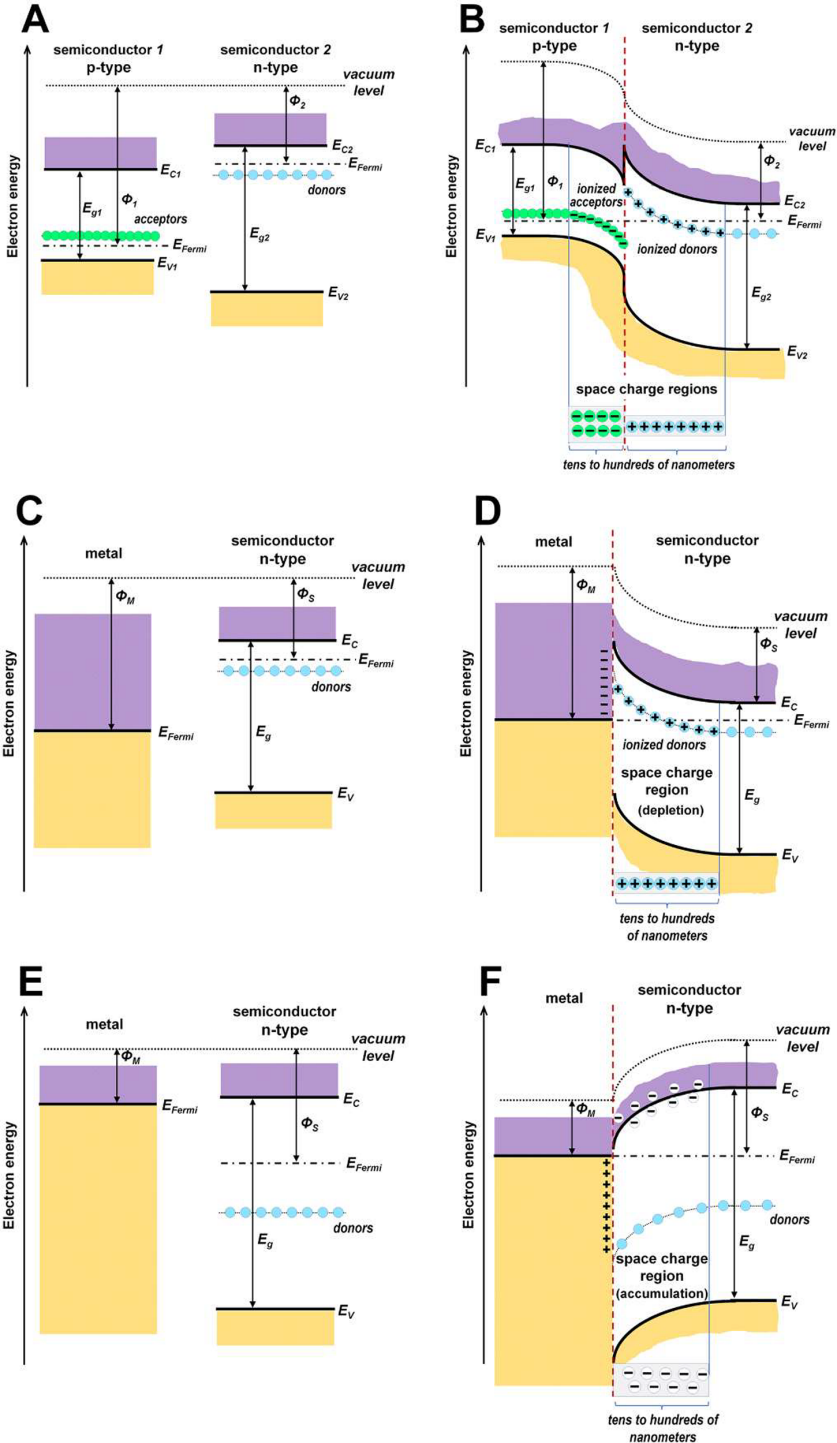
Examples of junction band diagrams. (A) p- and n-type semiconductors before contact and (B) after heterojunction formation. (C) Metal (with higher work function Φ_M_) and n-type semiconductor (with lower work function Φ_S_) before contact and (D) after formation of the Schottky barrier. (E) Metal and n-type semiconductor (in the case where Φ_M_ < Φ_S_) before contact and (F) after making an ohmic junction. (*E*_g_, band gap; *E*_Fermi_, Fermi level; and *E*_C_ and *E*_V_, lower edge of the conduction band and upper edge of the valence band, respectively).

The formation of the space charge on both sides of a heterojunction made of two semiconductors (depletion or accumulation regions) is a phenomenon that has been known for a long time, being, for example, the basis for the operation of light-emitting diodes or solar cells.^6^ When a simple heterojunction formed between p- and n-type semiconductors is taken as an example, its band diagram can be represented in panels A and B of Figure 1. As a result of the natural phenomenon of achieving thermodynamic equilibrium (equalization of the Fermi level), a positively charged region (ionized donors) and a negatively charged region (ionized acceptors) are formed on the n- and p-type sides, respectively. Of course, the presented heterojunction is only one of many possible combinations between dissimilar semiconductors with different band gaps, different types of conductivity, and different positions in the energy scale of the edges of the conduction and valence bands.^7^ In each of these cases, without exception, space charge regions are formed as ionized donors or acceptors (depletion regions) or electrons or holes placed in shallow traps (accumulation regions). The width of these space charge regions in many cases reaches even several hundred nanometers.^6^

An analogous situation, also known for a long time, can be achieved by creating junctions of metal with a semiconductor.^5,6^ When a n-type semiconductor with work function Φ_S_ and producing its junction with a metal having a higher work function Φ_M_ (Φ_M_ > Φ_S_) is taken as an example, a Schottky barrier is formed (panels C and D of Figure 1) with a region of positive space charge on the semiconductor side (depletion region with ionized donors). On the other hand, if Φ_M_ < Φ_S_, an ohmic junction is formed (panels E and F of Figure 1) with an accumulation region of negative charge (electrons in shallow traps). As in the case of semiconductor–semiconductor junctions, the width of the space charge region on the semiconductor side of metal–semiconductor junctions can also reach several hundred nanometers.

**Figure 2:**
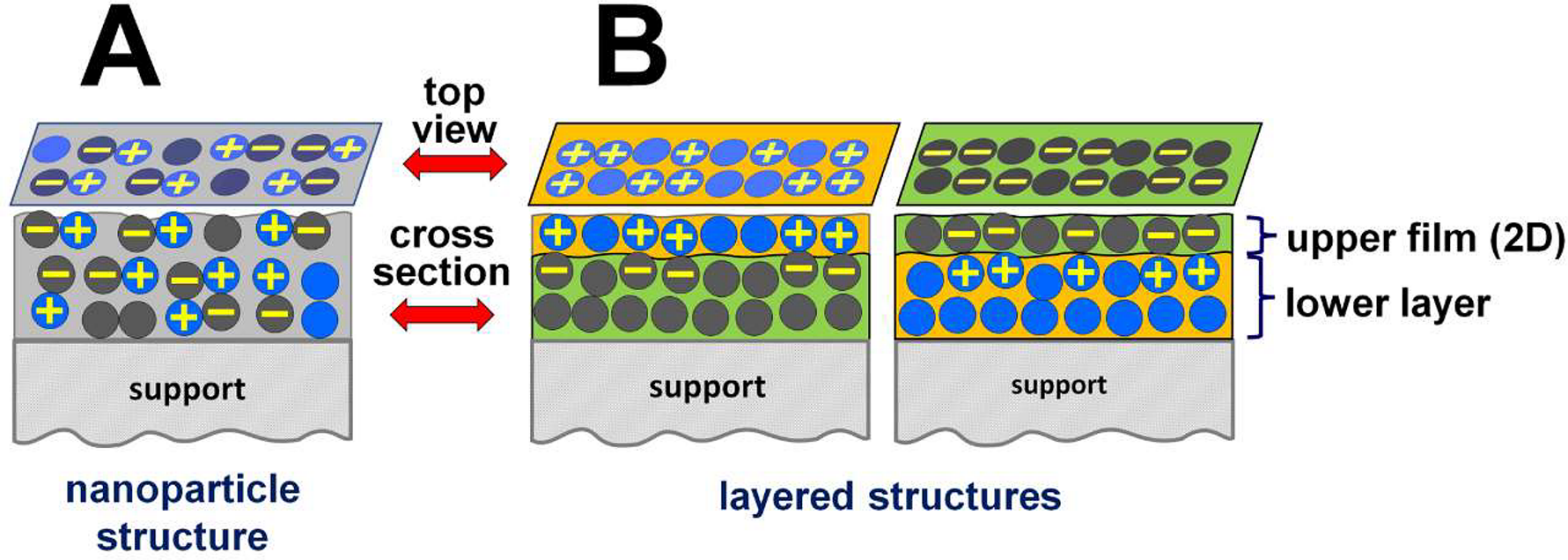
Sketch of two basic categories of semiconductor–semiconductor nanohybrids with heterojunctions illustrating the possibility of tailoring the catalytic properties of the surface. (A) Nanoparticle structure where the nanoparticles of both semiconductors (blue and gray) are mixed in bulk. (B) Two variants of the nanolayered structure where a thin film of one semiconductor is deposited on a layer of the other.

In nanohybrid catalysts, when the components in contact are nanometer-sized objects, the space charge regions can completely fill them, altering their electronic structure. Consequently, as was considered quite a long time ago,^8^ this can also change the electronic nature of the active sites on their surface, which is crucial in catalysis processes, because it directly influences the adsorption and activation of reactant molecules on these sites. Some even argue that without these electronic effects, there would be no catalysis.^9^ By fine-tuning the surface charge through the design of heterojunctions, we can manipulate the interaction between the nanohybrid catalyst and reactants, ultimately regulating the overall catalytic activity and selectivity. This modulation of catalytic activity by inducing charge transfer effects through heterojunctions can be achieved by designing nanocatalysts in two basic categories: those with a nanoparticle structure (Figure 2A) and those with a nanolayered structure (Figure 2B).

## Particle Nanohybrids with Heterojunctions

In the vast majority of cases, heterojunctions formed in nanohybrids composed of semiconductor–semiconductor or metal–semiconductor nanoparticles have been studied only in catalytic processes involving light, focusing mainly on water splitting. It is true that researchers dealing with this subject have recently taken into account the presence of space charge regions in heterojunctions, even designating them as a new type of junction and calling them S-scheme heterojunctions. However, the role of space charge has been reduced here only to the factor causing the generation of an electric field in which photoexcited hole–electron pairs are separated.^10^

On the other hand, consideration on the effect of depletion or accumulation regions on the catalytic properties of nanohybrids without the participation of light (thermal catalysis) and even more so in their relationship with the nature of active sites has thus far been marginal.^11−14^ This does not mean, however, that this problem is not interesting and important for further progress in the rational design of catalytic nanomaterials. There is no doubt that, in the formed heterojunctions, with the exception of neutral junctions, which is rather a theoretical case, charge transfer occurs.

The presence of space charge regions in nanoscale heterojunctions can be confirmed by X-ray photoelectron spectroscopy (XPS) studies. The binding energy determined in this way for an electron from a given energy level in an atom of a given element is the result of the interaction of this electron with other electrons in the environment and with the atomic nucleus. The appearance of an additional density of negative or positive charge causes a decrease or increase in the binding energy, respectively. The observation of a binding energy shift toward higher values for one of the nanohybrid components and at the same time a shift toward lower values for the other component compared to the binding energies for pure components is a clear proof of the presence of space charge regions resulting from the formation of heterojunctions.

The above effect was found, for example, in the case of nanoscale heterojunctions produced in the form of a core–shell from TiO_2_ and ZnIn_2_S_4_,^15^ obtained by decorating red phosphorus particles with WO_3_ nanorods,^16^ in a system of MoO_3_ nanoparticles in a NiO matrix,^17^ nanohybrids created by depositing atomically precise silver nanoclusters (which can be regarded as small band gap semiconductors) onto TiO_2_ nanoparticles,^18^ MoC nanoparticles embedded inside a nitrogen-doped carbon support,^13^ or Pr_*x*_Ce_1–*x*_O_2_ and PtCu nanoparticles.^14^ Only in the last two cases, an attempt was made to test the obtained nanomaterial in the process of thermal catalysis or electrocatalysis, respectively, showing the relationship between the increase in catalytic activity and the presence of space charge.

**Figure 3:**
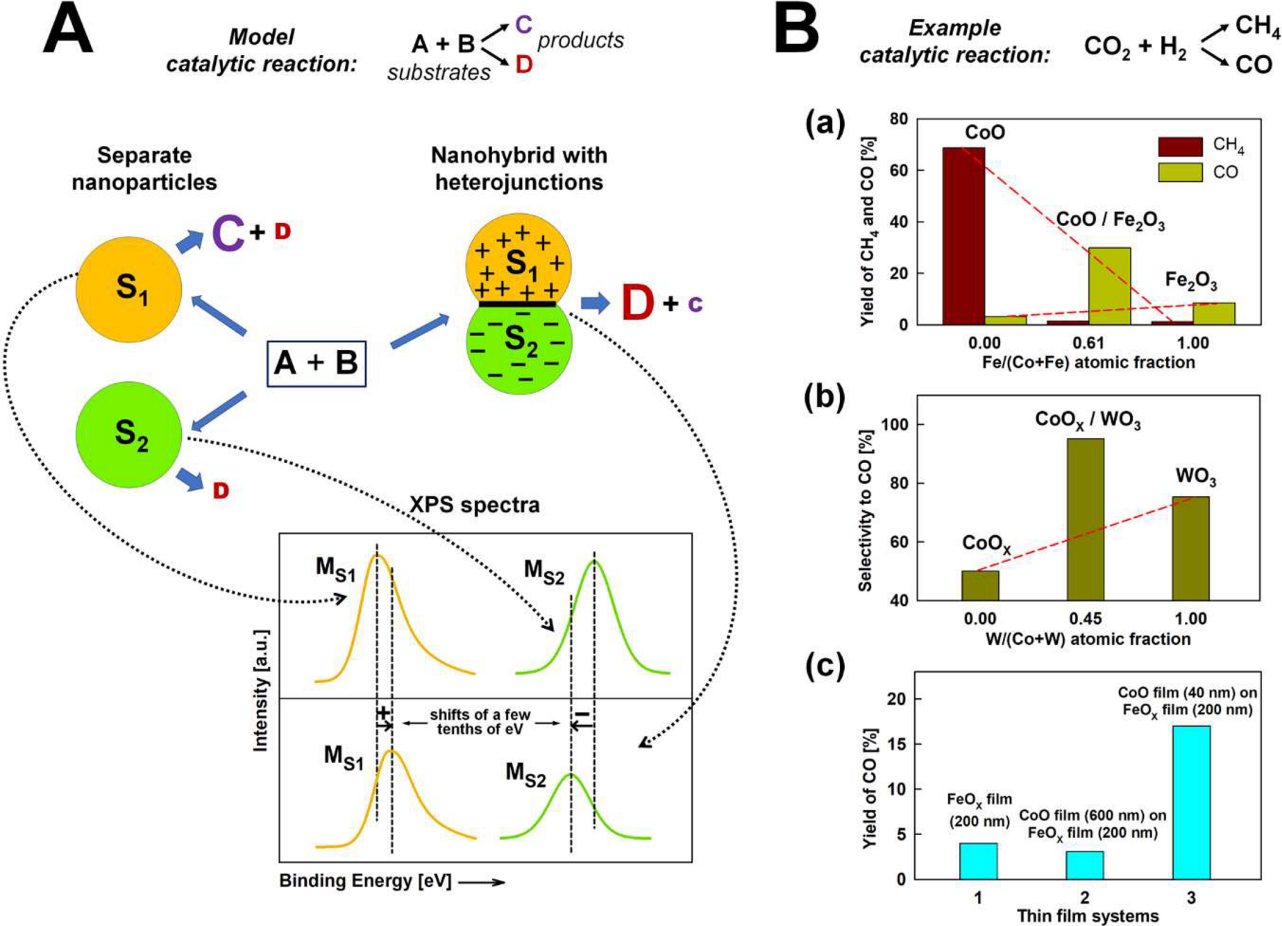
(A) Schematic model showing the role of nanoscale heterojunctions in the thermal catalysis process. Nanoparticles of two different semiconductors, S_1_ and S_2_, form a heterojunction. The space charges that arise and fill the nanoparticles cause shifts of the XPS bands for selected elements, M_S_1__ and M_S_2__. They also cause a change in the yield of the products, C and D, in the hypothetical reaction A + B compared to separately tested S_1_ and S_2_. (B) Selected results illustrating the role of nanoscale heterojunctions in controlling the activity in thermal catalysis processes on the example of CO_2_ hydrogenation: (a) yield of CH_4_ and CO depending upon the atomic composition of the CoO/Fe_2_O_3_ particle nanohybrid (on the basis of ref (19)), (b) selectivity to CO depending upon the atomic composition of the CoO_*x*_/WO_3_ particle nanohybrid, and (c) CO yield for the nanolayered system composed of CoO and FeO_*x*_ films. The red dashed lines, in panels a and b, confirm the non-additive nature of the dependence upon atomic compositions.

To better illustrate this phenomenon, its schematic model is presented in Figure 3A. Nanoparticles of two different semiconductors, S_1_ and S_2_, when forming a heterojunction generate a region of space charge that can permeate the entire volume of these nanoparticles. This is evident in the shifting of the XPS bands for the characteristic elements constituting these semiconductors (referred to as M_S_1__ and M_S_2__ for S_1_ and S_2_ semiconductors, respectively). Moreover, the presence of charge modifies the nature of active sites on the nanoparticle surface, leading to a change in catalytic activity in the hypothetical reaction, A + B. This change is manifested by a shift in the yield of a given product, C and D.

The confirmation that nanohybrids composed of nanoparticles of two different semiconductors, forming heterojunctions with space charge, exhibit differences in catalytic properties in thermal catalysis compared to independently tested nanoparticles of those semiconductors has been established in our recent studies. These studies were conducted on thin-film nanohybrids produced using the cold plasma deposition technique, including CoO/FeO_*x*_,^19^ Fe_2_O_3_/carbon nanotubes (CNTs),^20^ CoO/carbon matrix (CM),^21^ and currently investigated CoO_*x*_/WO_3_. The chosen catalytic process for these cases was the hydrogenation of CO_2_, which can proceed via two pathways to produce CO or CH_4_ as useful products. In these experiments, we observed band shifts in the XPS spectra of the nanohybrids in relation to the isolated components, indicating the presence of space charges. We also found a clear influence of the formed heterojunctions on the catalytic activity.

Panels a and b of Figure 3B present selected results from the above studies, illustrating the important role of nanoscale heterojunctions in controlling the activity in thermal catalysis processes. This includes the CO and CH_4_ yields depending upon the Fe/(Co + Fe) atomic fraction in the CoO/FeO_*x*_ nanohybrid and CO selectivity on the W/(Co + W) atomic fraction for the CoO_*x*_/WO_3_ nanohybrid. In both cases, the non-additive nature of the presented relationships confirms the occurrence of interactions between nanoparticles of two different semiconductors and attributes this phenomenon to the formation of heterojunctions. As a result, the nanoparticles are filled with a positive or negative space charge, dramatically changing their catalytic properties. Thus, by designing appropriate heterojunctions, we can direct the reaction along the chosen pathway toward CO or CH_4_.

## Nanolayered Systems with Heterojunctions

If we prepare a system (schematically shown in Figure 2B), wherein an appropriately thin [two-dimensional (2D)] film of a semiconductor is deposited on another semiconductor layer, the creation of a heterojunction will result in the 2D film being filled with a positive or negative space charge, thereby modifying the catalytic properties of its surface (this consideration can, of course, also be adapted for the case of metal–semiconductor junctions, as shown in panels D or F of Figure 1). By selecting the type and electronic structure of the materials for the lower and upper (2D) layers, we can control the electronic properties and, thus, the catalytic activity of the 2D film surface.

While this assumption may seem obvious, the influence of the support on the catalytic properties of the material deposited on it in the form of a thin film (or a similar system in which the deposited material consists of discrete nanoparticles) is often explained in a complex, sophisticated manner, overlooking the simple formation of heterojunctions with depletion or accumulation regions.^22^ This approach could, for instance, easily explain the increase in the shift of the XPS Pt 4f_7/2_ band maximum for Pt nanoparticles grown on CeO_*x*_ with a decrease in the size of these nanoparticles, as well as the effect of this size on the average charge accumulated in the Pt nanoparticle.^23,24^ As the Pt nanoparticle size decreases, the average charge increases (up to a certain limit), consequently resulting in a greater Pt 4f_7/2_ band shift.

The phenomenon of creating a space charge region throughout the upper film in nanolayered systems is still awaiting recognition in thermal catalysis. Perhaps this concept could offer more rational insight into explaining various catalytic processes. For instance, it could elucidate the catalytic activity in CO oxidation over a catalyst composed of ZrO_2_ fibers and an atomic layer deposition (ALD)-deposited thin film of CoO_*x*_.^25^ Additionally, it could provide a deeper understanding of the influence of Al doping in a ZnO semiconductor, forming a junction with Cu, on the catalytic activity of CO formation in the reverse water–gas shift reaction.^26^ Furthermore, considering the Pt nanoparticles on CeO_*x*_ mentioned earlier, this concept could offer a fresh perspective on catalytic decomposition processes in automobile exhaust gases.

With the idea of managing the type and density of charge in a thin film solely through a proper heterojunction with another material put aside, attempts have been made to control this charge by applying an electric voltage between the support and the upper thin film. Changing the voltage value and its direction allows for the manipulation of the sign and charge density on the surface of the catalyst nanolayered system, thereby tuning its catalytic chemistry.^27^ Although this appears to be a terrific solution, its implementation on a wider industrial scale, such as in the manufacture of structured packings for catalytic reactors, raises serious challenges. It seems that, in many circumstances, constructing appropriate heterojunctions using thin-film materials with a suitably tuned electronic structure would be a simpler and sufficient method of designing catalytic capabilities.

The initial results that we have just obtained in the field of nanolayered systems are already highly encouraging from the perspective of controlling catalytic properties by inducing charge transfer effects through heterojunctions. Three thin-film catalyst systems prepared by cold plasma deposition were compared in the hydrogenation reaction of CO_2_ to CO (panel c of Figure 3B): (1) a single FeO_*x*_-based layer (thickness of ≈200 nm) and CoO-based films with thicknesses of (2) ≈600 nm and (3) ≈40 nm deposited on top of the iron oxide layer. A significant increase in the CO yield is visible for the thin CoO-based film compared to the thick film. This change in yield aligns with predictions based on the behavior of the heterojunction between CoO and FeO_*x*_, of which the effect is evident on the surface of the thin CoO-based film but is no longer observed on the surface of the thick film.

## Cold Plasma Deposition—A Great Tool for Creating Nanocatalysts with Heterojunctions

One of the extremely promising methods to be offered for the simple and large-scale fabrication of nanohybrids with heterojunctions of both particle and nanolayer types is the cold plasma deposition approach, often referred to as plasma-enhanced chemical vapor deposition (PECVD). The PECVD technique has long been employed to create new materials with unique properties, of which the vast majority cannot be produced by other methods. Among the many interesting materials synthesized by this technique, a special place is occupied by very thin films with catalytic properties, which have recently garnered increasing attention.^28−30^

In the PECVD method, to simplify, films are deposited from volatile precursors, mainly organic or organometallic, fed under reduced pressure into a reactor where non-equilibrium plasma (also often called cold plasma) is generated by an electrical (glow) discharge. This plasma can be generated in various ways, starting with a direct current (DC) discharge, through kilohertz (kHz) and megahertz (usually 13.56 MHz) frequencies, and ending with a microwave discharge. The ability to choose the chemical structure of the precursor, utilize a mixture of two or more precursors, modify the composition of the reaction mixture during the deposition process, and control a wide range of process parameters, such as the type and power of the discharge, flow rate of the reaction mixture components through the reactor, temperature, etc., provides significant opportunities to tailor the molecular structure and nanostructure of the films. This allows for the fabrication of designed nanosystems.^31^

In this manner, we can control the type, density, and size of nanoparticles as well as the thickness of nanolayers in the produced nanohybrids and, thus, also the type of heterojunctions and their electronic structure, offering ample room for exploration in the quest for appropriate catalytic activity. Nanohybrids with heterojunctions of the nanoparticle and nanolayered types produced by the PECVD technique have already been demonstrated above, including in the examples shown in Figure 3B.

When the PECVD technique is discussed, another crucial aspect should be noted: the ability to deposit the produced materials on virtually any support, regardless of the shape and size, without altering the geometry of the support as a result of the very small thickness (ranging from nano- to micrometers). It is difficult to envision further progress in the construction of catalytic structured reactors without very thin catalytic films, which can be easily and inexpensively deposited on precisely designed, sophisticated packing. No other method can rival PECVD in this regard.

## Outlook

Realizing that two nanometer-sized elements from different semiconductors can form a heterojunction after contact with each other and can become completely filled with space charge resulting from the formation of depletion or accumulation regions opens up the possibility of designing the electronic structure of the surface of nanohybrids composed of such elements. This approach can be used to regulate catalytic activity.

By controlling the surface charge of the nanohybrid catalyst through the appropriate structure of heterojunctions, we can enhance its catalytic activity, which will differ significantly from the additive behavior of the components forming such a nanohybrid. Of course, many details regarding the design of nanohybrids with heterojunctions require further advanced research and deeper analysis, especially when considering nanoscale objects and their electronic structure, which may differ substantially from the electronic structure of such materials in the much better known to us macroscale. Nevertheless, the concept of heterojunctions on the nanoscale and the control of the activity and selectivity of nanohybrids in thermal catalysis processes have solid foundations. The recent experimental findings presented here support the validity of this idea, an idea that offers a new perspective for the contemporary “alchemy” of nanocatalysts.

If we add the huge advantages of the PECVD method, first, the ease of controlling the molecular structure and nanostructure of the produced nanohybrids over a wide range (including co-deposition from a mixture of various precursors, layer-on-layer deposition, plasma treatment, and functionalization of the surface of the deposited films) and, second, the production of the catalytic material in the form of very thin films, which are invaluable in the development of structured catalytic reactors, we will be able to transfer the proposed idea based on nanohybrids with heterojunctions from theoretical considerations and basic research to practical applications.
